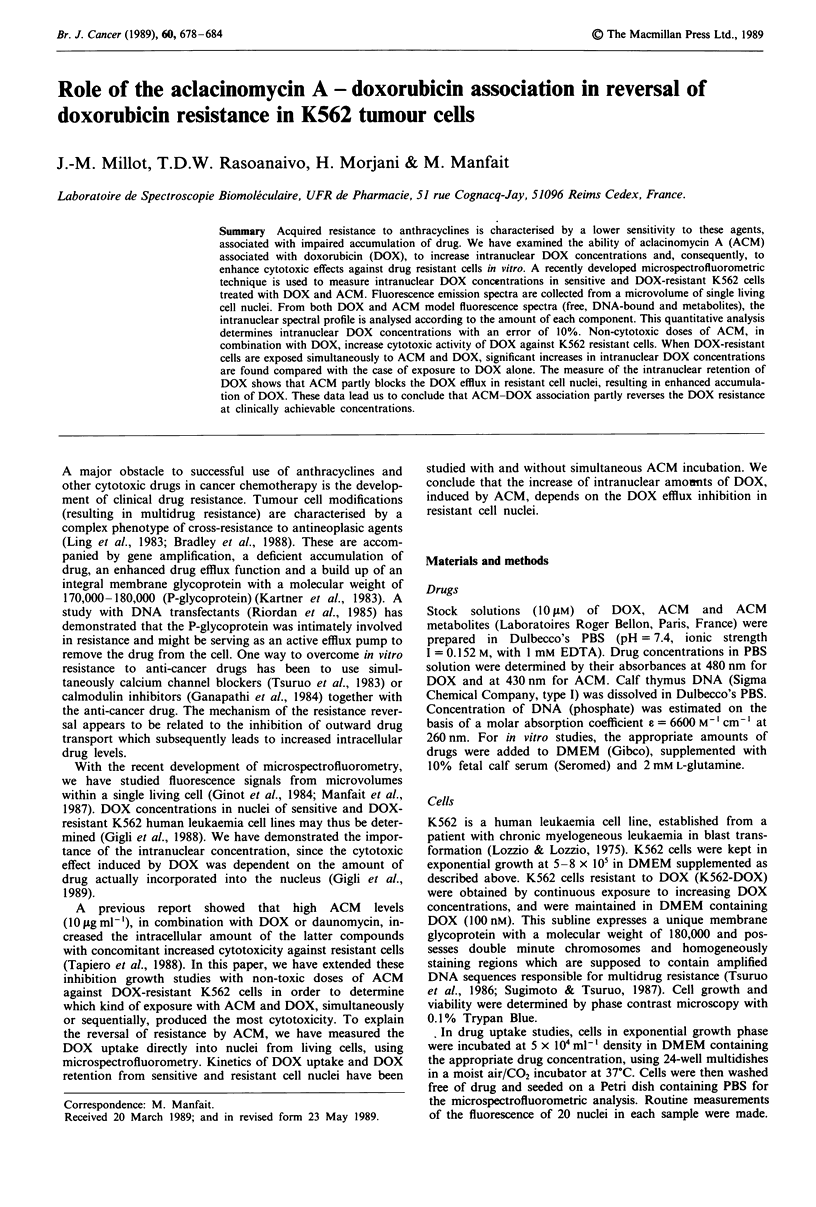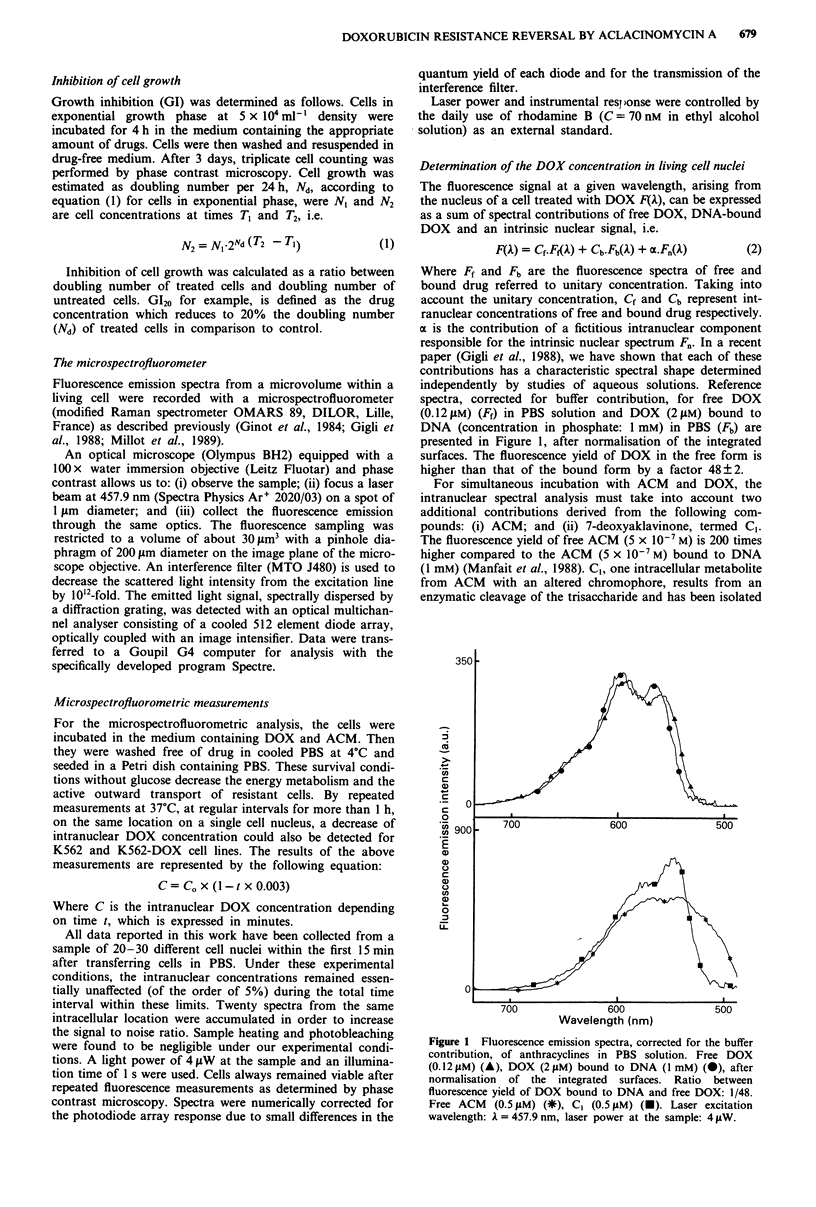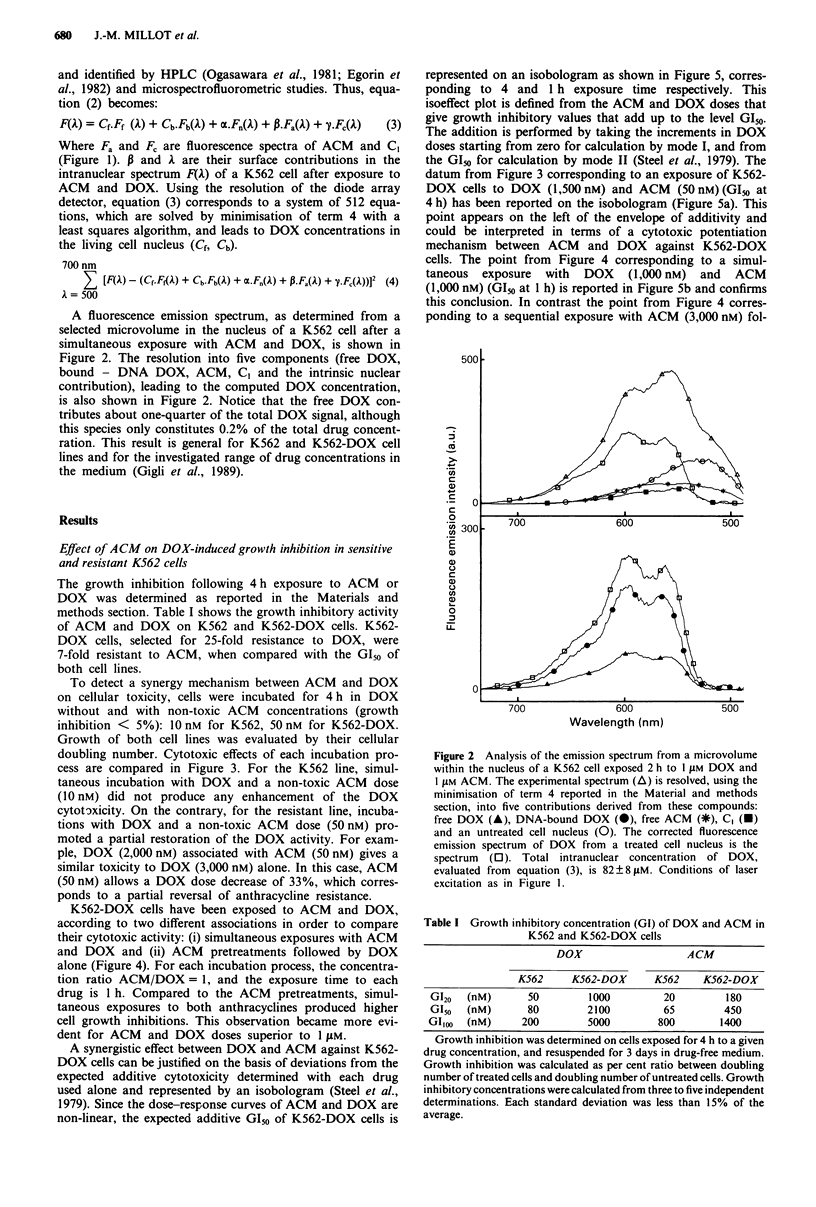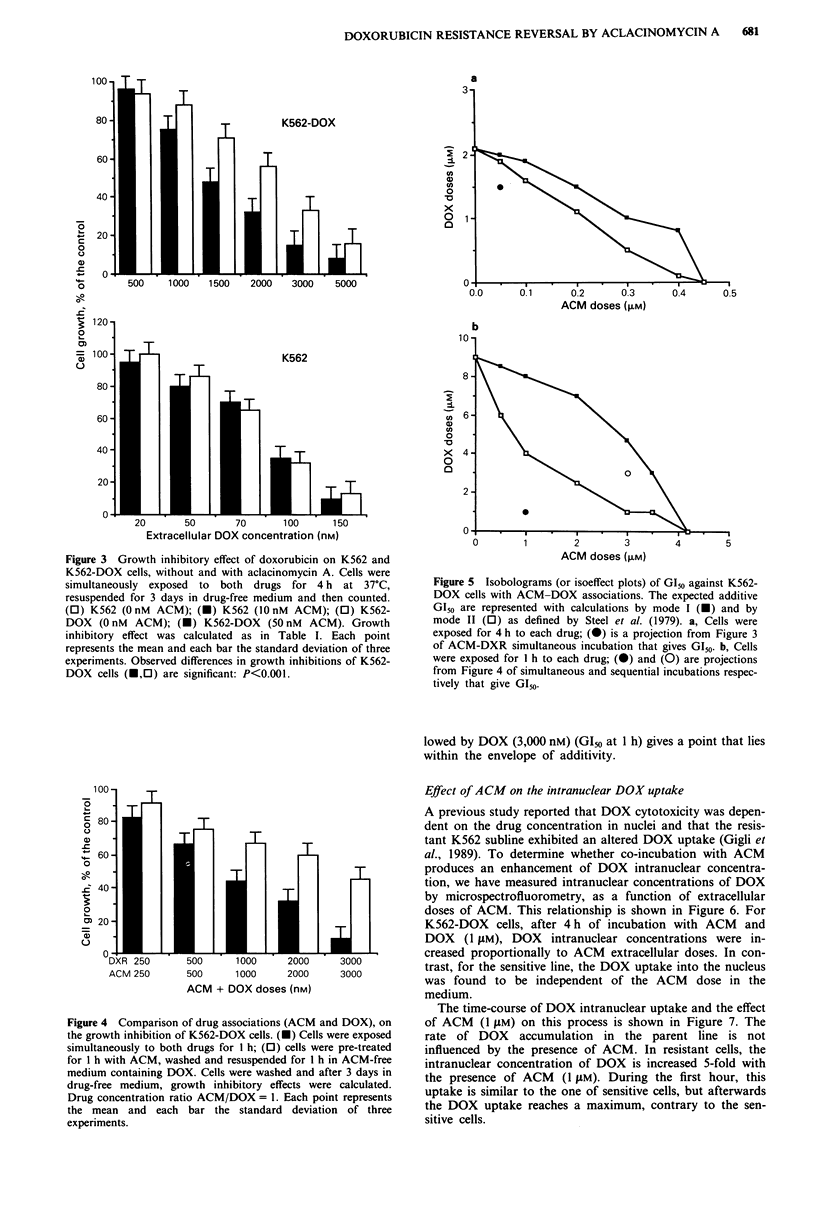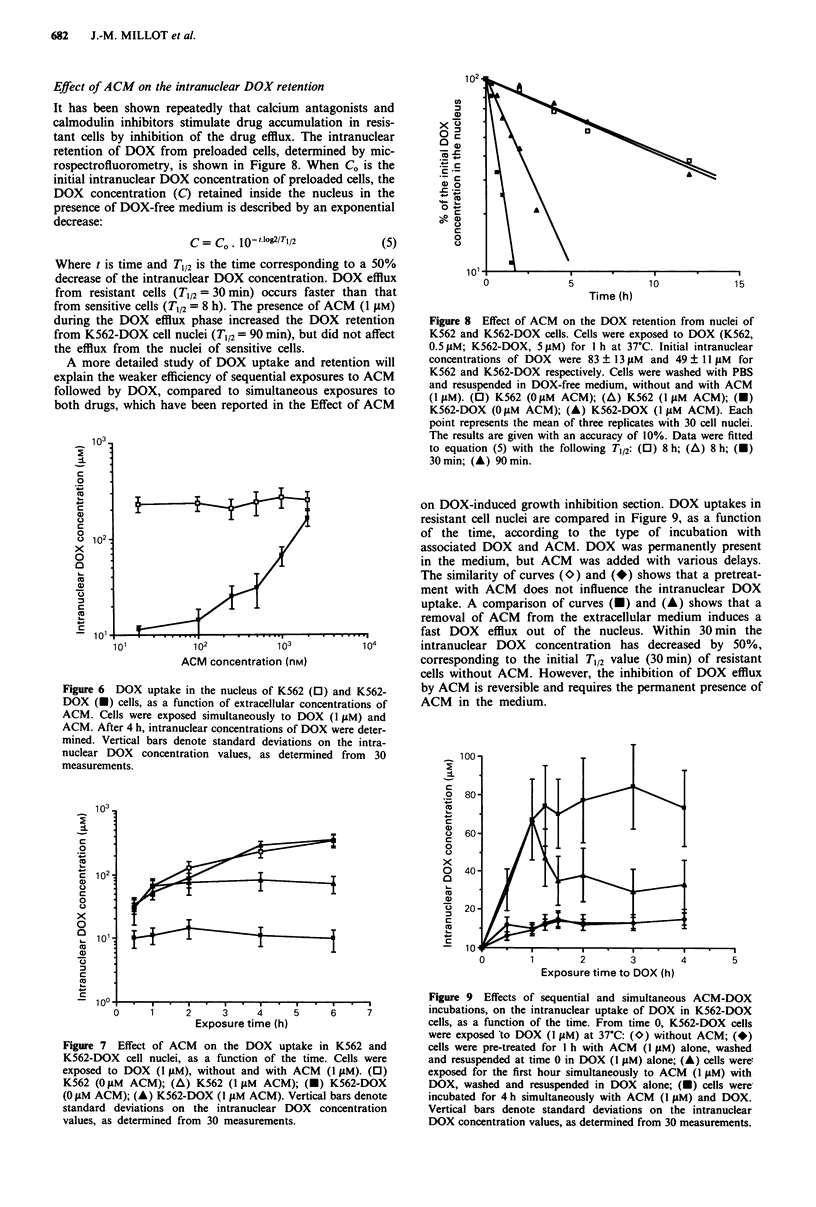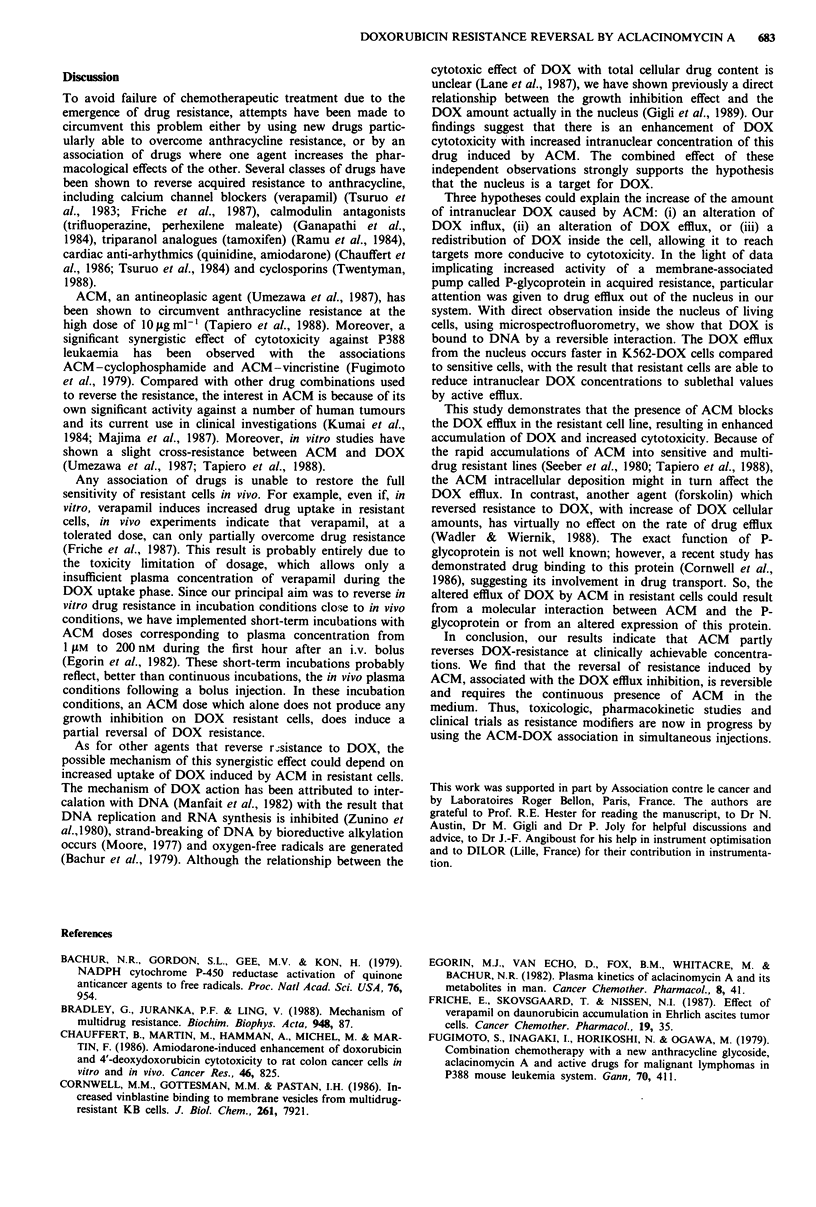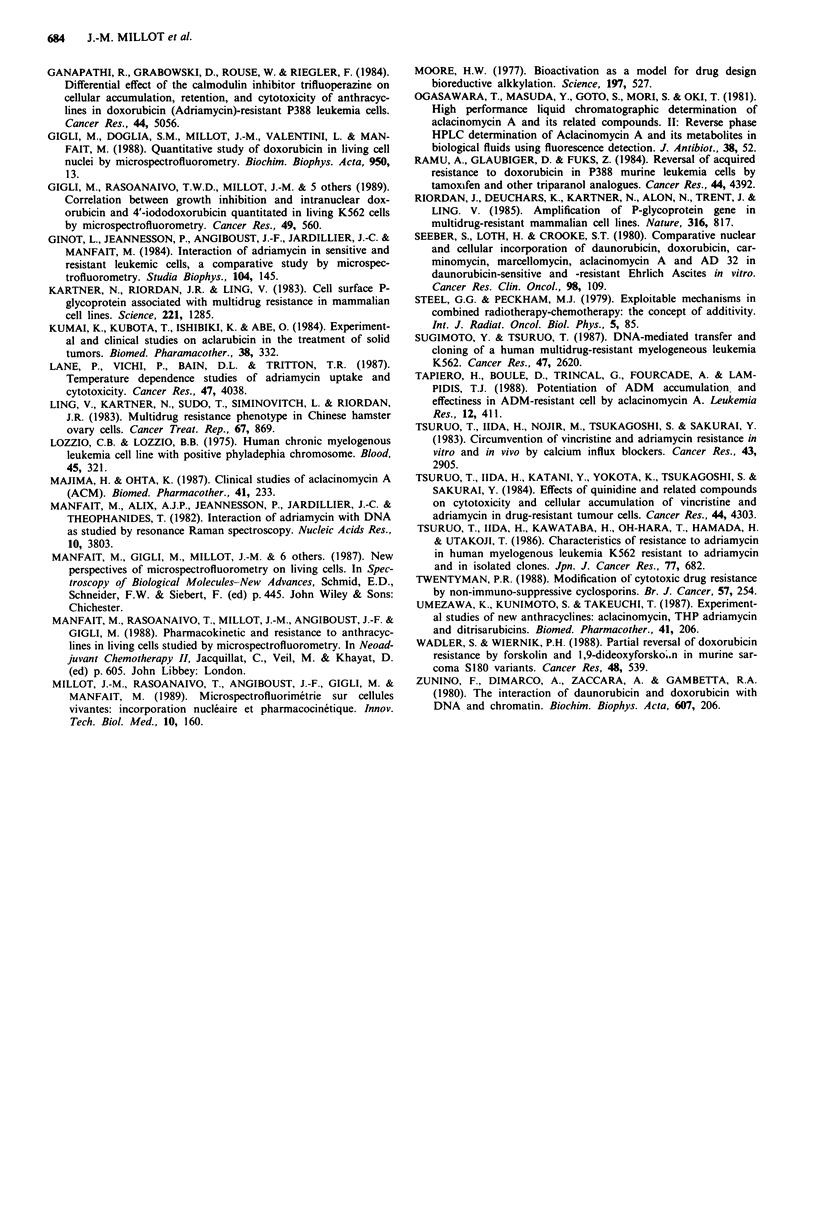# Role of the aclacinomycin A--doxorubicin association in reversal of doxorubicin resistance in K562 tumour cells.

**DOI:** 10.1038/bjc.1989.339

**Published:** 1989-11

**Authors:** J. M. Millot, T. D. Rasoanaivo, H. Morjani, M. Manfait

**Affiliations:** Laboratoire de Spectroscopie BiomolÃ©culaire, UFR de Pharmacie, Reims, France.

## Abstract

Acquired resistance to anthracyclines is characterised by a lower sensitivity to these agents, associated with impaired accumulation of drug. We have examined the ability of aclacinomycin A (ACM) associated with doxorubicin (DOX), to increase intranuclear DOX concentrations and, consequently, to enhance cytotoxic effects against drug resistant cells in vitro. A recently developed microspectrofluorometric technique is used to measure intranuclear DOX concentrations in sensitive and DOX-resistant K562 cells treated with DOX and ACM. Fluorescence emission spectra are collected from a microvolume of single living cell nuclei. From both DOX and ACM model fluorescence spectra (free, DNA-bound and metabolites), the intranuclear spectral profile is analysed according to the amount of each component. This quantitative analysis determines intranuclear DOX concentrations with an error of 10%. Non-cytotoxic doses of ACM, in combination with DOX, increase cytotoxic activity of DOX against K562 resistant cells. When DOX-resistant cells are exposed simultaneously to ACM and DOX, significant increases in intranuclear DOX concentrations are found compared with the case of exposure to DOX alone. The measure of the intranuclear retention of DOX shows that ACM partly blocks the DOX efflux in resistant cell nuclei, resulting in enhanced accumulation of DOX. These data lead us to conclude that ACM-DOX association partly reverses the DOX resistance at clinically achievable concentrations.


*Br. J. Cancer* (1989), **60**, 678–684 © The Macmillan Press Ltd., 1989

### Role of the aclacinomycin A – doxorubicin association in reversal of doxorubicin resistance in K562 tumour cells

J.-M. Millot, T.D.W. Rasoanaivo, H. Morjani & M. Manfait

*Laboratoire de Spectroscopie Biomoléculaire, UFR de Pharmacie, 51 rue Cognacq-Jay, 51096 Reims Cedex, France.*

**Summary** Acquired resistance to anthracyclines is characterised by a lower sensitivity to these agents, associated with impaired accumulation of drug. We have examined the ability of aclacinomycin A (ACM) associated with doxorubicin (DOX), to increase intranuclear DOX concentrations and, consequently, to enhance cytotoxic effects against drug resistant cells *in vitro*. A recently developed microspectrofluorometric technique is used to measure intranuclear DOX concentrations in sensitive and DOX-resistant K562 cells treated with DOX and ACM. Fluorescence emission spectra are collected from a microvolume of single living cell nuclei. From both DOX and ACM model fluorescence spectra (free, DNA-bound and metabolites), the intranuclear spectral profile is analysed according to the amount of each component. This quantitative analysis determines intranuclear DOX concentrations with an error of 10%. Non-cytotoxic doses of ACM, in combination with DOX, increase cytotoxic activity of DOX against K562 resistant cells. When DOX-resistant cells are exposed simultaneously to ACM and DOX, significant increases in intranuclear DOX concentrations are found compared with the case of exposure to DOX alone. The measure of the intranuclear retention of DOX shows that ACM partly blocks the DOX efflux in resistant cell nuclei, resulting in enhanced accumulation of DOX. These data lead us to conclude that ACM–DOX association partly reverses the DOX resistance at clinically achievable concentrations.

A major obstacle to successful use of anthracyclines and other cytotoxic drugs in cancer chemotherapy is the development of clinical drug resistance. Tumour cell modifications (resulting in multidrug resistance) are characterised by a complex phenotype of cross-resistance to antineoplasic agents (Ling *et al.*, 1983; Bradley *et al.*, 1988). These are accompanied by gene amplification, a deficient accumulation of drug, an enhanced drug efflux function and a build up of an integral membrane glycoprotein with a molecular weight of 170,000–180,000 (P-glycoprotein) (Kartner *et al.*, 1983). A study with DNA transfectants (Riordan *et al.*, 1985) has demonstrated that the P-glycoprotein was intimately involved in resistance and might be serving as an active efflux pump to remove the drug from the cell. One way to overcome *in vitro* resistance to anti-cancer drugs has been to use simultaneously calcium channel blockers (Tsuruo *et al.*, 1983) or calmodulin inhibitors (Ganapathi *et al.*, 1984) together with the anti-cancer drug. The mechanism of the resistance reversal appears to be related to the inhibition of outward drug transport which subsequently leads to increased intracellular drug levels.

With the recent development of microspectrofluorometry, we have studied fluorescence signals from microvolumes within a single living cell (Ginot *et al.*, 1984; Manfait *et al.*, 1987). DOX concentrations in nuclei of sensitive and DOX-resistant K562 human leukaemia cell lines may thus be determined (Gigli *et al.*, 1988). We have demonstrated the importance of the intranuclear concentration, since the cytotoxic effect induced by DOX was dependent on the amount of drug actually incorporated into the nucleus (Gigli *et al.*, 1989).

A previous report showed that high ACM levels ($10\,\mu g\,ml^{-1}$), in combination with DOX or daunomycin, increased the intracellular amount of the latter compounds with concomitant increased cytotoxicity against resistant cells (Tapiero *et al.*, 1988). In this paper, we have extended these inhibition growth studies with non-toxic doses of ACM against DOX-resistant K562 cells in order to determine which kind of exposure with ACM and DOX, simultaneously or sequentially, produced the most cytotoxicity. To explain the reversal of resistance by ACM, we have measured the DOX uptake directly into nuclei from living cells, using microspectrofluorometry. Kinetics of DOX uptake and DOX retention from sensitive and resistant cell nuclei have been studied with and without simultaneous ACM incubation. We conclude that the increase of intranuclear amounts of DOX, induced by ACM, depends on the DOX efflux inhibition in resistant cell nuclei.

Correspondence: M. Manfait.
Received 20 March 1989; and in revised form 23 May 1989.

### Materials and methods

### *Drugs*

Stock solutions ($10\,\mu$M) of DOX, ACM and ACM metabolites (Laboratoires Roger Bellon, Paris, France) were prepared in Dulbecco's PBS (pH = 7.4, ionic strength I = 0.152 M, with 1 mM EDTA). Drug concentrations in PBS solution were determined by their absorbances at 480 nm for DOX and at 430 nm for ACM. Calf thymus DNA (Sigma Chemical Company, type I) was dissolved in Dulbecco's PBS. Concentration of DNA (phosphate) was estimated on the basis of a molar absorption coefficient $\varepsilon = 6600\,M^{-1}\,cm^{-1}$ at 260 nm. For *in vitro* studies, the appropriate amounts of drugs were added to DMEM (Gibco), supplemented with 10% fetal calf serum (Seromed) and 2 mM L-glutamine.

### *Cells*

K562 is a human leukaemia cell line, established from a patient with chronic myelogeneous leukaemia in blast transformation (Lozzio & Lozzio, 1975). K562 cells were kept in exponential growth at $5–8 \times 10^5$ in DMEM supplemented as described above. K562 cells resistant to DOX (K562-DOX) were obtained by continuous exposure to increasing DOX concentrations, and were maintained in DMEM containing DOX (100 nM). This subline expresses a unique membrane glycoprotein with a molecular weight of 180,000 and possesses double minute chromosomes and homogeneously staining regions which are supposed to contain amplified DNA sequences responsible for multidrug resistance (Tsuruo *et al.*, 1986; Sugimoto & Tsuruo, 1987). Cell growth and viability were determined by phase contrast microscopy with 0.1% Trypan Blue.

In drug uptake studies, cells in exponential growth phase were incubated at $5 \times 10^4\,ml^{-1}$ density in DMEM containing the appropriate drug concentration, using 24-well multidishes in a moist air/$CO_2$ incubator at 37°C. Cells were then washed free of drug and seeded on a Petri dish containing PBS for the microspectrofluorometric analysis. Routine measurements of the fluorescence of 20 nuclei in each sample were made.

*Inhibition of cell growth*

Growth inhibition (GI) was determined as follows. Cells in exponential growth phase at $5 \times 10^4\,ml^{-1}$ density were incubated for 4 h in the medium containing the appropriate amount of drugs. Cells were then washed and resuspended in drug-free medium. After 3 days, triplicate cell counting was performed by phase contrast microscopy. Cell growth was estimated as doubling number per 24 h, $N_d$, according to equation (1) for cells in exponential phase, were $N_1$ and $N_2$ are cell concentrations at times $T_1$ and $T_2$, i.e.

$$N_2 = N_1 \cdot 2^{N_d}(T_2 - T_1) \qquad (1)$$

Inhibition of cell growth was calculated as a ratio between doubling number of treated cells and doubling number of untreated cells. $GI_{20}$ for example, is defined as the drug concentration which reduces to 20% the doubling number ($N_d$) of treated cells in comparison to control.

*The microspectrofluorometer*

Fluorescence emission spectra from a microvolume within a living cell were recorded with a microspectrofluorometer (modified Raman spectrometer OMARS 89, DILOR, Lille, France) as described previously (Ginot *et al.*, 1984; Gigli *et al.*, 1988; Millot *et al.*, 1989).

An optical microscope (Olympus BH2) equipped with a 100× water immersion objective (Leitz Fluotar) and phase contrast allows us to: (i) observe the sample; (ii) focus a laser beam at 457.9 nm (Spectra Physics $Ar^+$ 2020/03) on a spot of 1 μm diameter; and (iii) collect the fluorescence emission through the same optics. The fluorescence sampling was restricted to a volume of about 30 $\mu m^3$ with a pinhole diaphragm of 200 μm diameter on the image plane of the microscope objective. An interference filter (MTO J480) is used to decrease the scattered light intensity from the excitation line by $10^{12}$-fold. The emitted light signal, spectrally dispersed by a diffraction grating, was detected with an optical multichannel analyser consisting of a cooled 512 element diode array, optically coupled with an image intensifier. Data were transferred to a Goupil G4 computer for analysis with the specifically developed program Spectre.

*Microspectrofluorometric measurements*

For the microspectrofluorometric analysis, the cells were incubated in the medium containing DOX and ACM. Then they were washed free of drug in cooled PBS at 4°C and seeded in a Petri dish containing PBS. These survival conditions without glucose decrease the energy metabolism and the active outward transport of resistant cells. By repeated measurements at 37°C, at regular intervals for more than 1 h, on the same location on a single cell nucleus, a decrease of intranuclear DOX concentration could also be detected for K562 and K562-DOX cell lines. The results of the above measurements are represented by the following equation:

$$C = C_o \times (1 - t \times 0.003)$$

Where $C$ is the intranuclear DOX concentration depending on time $t$, which is expressed in minutes.

All data reported in this work have been collected from a sample of 20–30 different cell nuclei within the first 15 min after transferring cells in PBS. Under these experimental conditions, the intranuclear concentrations remained essentially unaffected (of the order of 5%) during the total time interval within these limits. Twenty spectra from the same intracellular location were accumulated in order to increase the signal to noise ratio. Sample heating and photobleaching were found to be negligible under our experimental conditions. A light power of 4 μW at the sample and an illumination time of 1 s were used. Cells always remained viable after repeated fluorescence measurements as determined by phase contrast microscopy. Spectra were numerically corrected for the photodiode array response due to small differences in the quantum yield of each diode and for the transmission of the interference filter.

Laser power and instrumental response were controlled by the daily use of rhodamine B ($C$ = 70 nM in ethyl alcohol solution) as an external standard.

*Determination of the DOX concentration in living cell nuclei*

The fluorescence signal at a given wavelength, arising from the nucleus of a cell treated with DOX $F(\lambda)$, can be expressed as a sum of spectral contributions of free DOX, DNA-bound DOX and an intrinsic nuclear signal, i.e.

$$F(\lambda) = C_f.F_f(\lambda) + C_b.F_b(\lambda) + \alpha.F_n(\lambda) \qquad (2)$$

Where $F_f$ and $F_b$ are the fluorescence spectra of free and bound drug referred to unitary concentration. Taking into account the unitary concentration, $C_f$ and $C_b$ represent intranuclear concentrations of free and bound drug respectively. $\alpha$ is the contribution of a fictitious intranuclear component responsible for the intrinsic nuclear spectrum $F_n$. In a recent paper (Gigli *et al.*, 1988), we have shown that each of these contributions has a characteristic spectral shape determined independently by studies of aqueous solutions. Reference spectra, corrected for buffer contribution, for free DOX (0.12 μM) ($F_f$) in PBS solution and DOX (2 μM) bound to DNA (concentration in phosphate: 1 mM) in PBS ($F_b$) are presented in Figure 1, after normalisation of the integrated surfaces. The fluorescence yield of DOX in the free form is higher than that of the bound form by a factor 48±2.

For simultaneous incubation with ACM and DOX, the intranuclear spectral analysis must take into account two additional contributions derived from the following compounds: (i) ACM; and (ii) 7-deoxyaklavinone, termed $C_1$. The fluorescence yield of free ACM ($5 \times 10^{-7}$ M) is 200 times higher compared to the ACM ($5 \times 10^{-7}$ M) bound to DNA (1 mM) (Manfait *et al.*, 1988). $C_1$, one intracellular metabolite from ACM with an altered chromophore, results from an enzymatic cleavage of the trisaccharide and has been isolated

**Figure 1** Fluorescence emission spectra, corrected for the buffer contribution, of anthracyclines in PBS solution. Free DOX (0.12 μM) (▲), DOX (2 μM) bound to DNA (1 mM) (●), after normalisation of the integrated surfaces. Ratio between fluorescence yield of DOX bound to DNA and free DOX: 1/48. Free ACM (0.5 μM) (✱), $C_1$ (0.5 μM) (■). Laser excitation wavelength: λ = 457.9 nm, laser power at the sample: 4 μW.

and identified by HPLC (Ogasawara *et al.*, 1981; Egorin *et al.*, 1982) and microspectrofluorometric studies. Thus, equation (2) becomes:

$$F(\lambda) = C_f.F_f\ (\lambda) + C_b.F_b(\lambda) + \alpha.F_n(\lambda) + \beta.F_a(\lambda) + \gamma.F_c(\lambda) \quad (3)$$

Where $F_a$ and $F_c$ are fluorescence spectra of ACM and $C_l$ (Figure 1). $\beta$ and $\lambda$ are their surface contributions in the intranuclear spectrum $F(\lambda)$ of a K562 cell after exposure to ACM and DOX. Using the resolution of the diode array detector, equation (3) corresponds to a system of 512 equations, which are solved by minimisation of term 4 with a least squares algorithm, and leads to DOX concentrations in the living cell nucleus ($C_f$, $C_b$).

$$\sum_{\lambda = 500}^{700\ \mathrm{nm}} [F(\lambda) - (C_f.F_f(\lambda) + C_b.F_b(\lambda) + \alpha.F_n(\lambda) + \beta.F_a(\lambda) + \gamma.F_c(\lambda))]^2 \quad (4)$$

A fluorescence emission spectrum, as determined from a selected microvolume in the nucleus of a K562 cell after a simultaneous exposure with ACM and DOX, is shown in Figure 2. The resolution into five components (free DOX, bound - DNA DOX, ACM, $C_l$ and the intrinsic nuclear contribution), leading to the computed DOX concentration, is also shown in Figure 2. Notice that the free DOX contributes about one-quarter of the total DOX signal, although this species only constitutes 0.2% of the total drug concentration. This result is general for K562 and K562-DOX cell lines and for the investigated range of drug concentrations in the medium (Gigli *et al.*, 1989).

### Results

### *Effect of ACM on DOX-induced growth inhibition in sensitive and resistant K562 cells*

The growth inhibition following 4 h exposure to ACM or DOX was determined as reported in the Materials and methods section. Table I shows the growth inhibitory activity of ACM and DOX on K562 and K562-DOX cells. K562-DOX cells, selected for 25-fold resistance to DOX, were 7-fold resistant to ACM, when compared with the $GI_{50}$ of both cell lines.

To detect a synergy mechanism between ACM and DOX on cellular toxicity, cells were incubated for 4 h in DOX without and with non-toxic ACM concentrations (growth inhibition $<$ 5%): 10 nM for K562, 50 nM for K562-DOX. Growth of both cell lines was evaluated by their cellular doubling number. Cytotoxic effects of each incubation process are compared in Figure 3. For the K562 line, simultaneous incubation with DOX and a non-toxic ACM dose (10 nM) did not produce any enhancement of the DOX cytotoxicity. On the contrary, for the resistant line, incubations with DOX and a non-toxic ACM dose (50 nM) promoted a partial restoration of the DOX activity. For example, DOX (2,000 nM) associated with ACM (50 nM) gives a similar toxicity to DOX (3,000 nM) alone. In this case, ACM (50 nM) allows a DOX dose decrease of 33%, which corresponds to a partial reversal of anthracycline resistance.

K562-DOX cells have been exposed to ACM and DOX, according to two different associations in order to compare their cytotoxic activity: (i) simultaneous exposures with ACM and DOX and (ii) ACM pretreatments followed by DOX alone (Figure 4). For each incubation process, the concentration ratio ACM/DOX = 1, and the exposure time to each drug is 1 h. Compared to the ACM pretreatments, simultaneous exposures to both anthracyclines produced higher cell growth inhibitions. This observation became more evident for ACM and DOX doses superior to 1 $\mu$M.

A synergistic effect between DOX and ACM against K562-DOX cells can be justified on the basis of deviations from the expected additive cytotoxicity determined with each drug used alone and represented by an isobologram (Steel *et al.*, 1979). Since the dose–response curves of ACM and DOX are non-linear, the expected additive $GI_{50}$ of K562-DOX cells is represented on an isobologram as shown in Figure 5, corresponding to 4 and 1 h exposure time respectively. This isoeffect plot is defined from the ACM and DOX doses that give growth inhibitory values that add up to the level $GI_{50}$. The addition is performed by taking the increments in DOX doses starting from zero for calculation by mode I, and from the $GI_{50}$ for calculation by mode II (Steel *et al.*, 1979). The datum from Figure 3 corresponding to an exposure of K562-DOX cells to DOX (1,500 nM) and ACM (50 nM) ($GI_{50}$ at 4 h) has been reported on the isobologram (Figure 5a). This point appears on the left of the envelope of additivity and could be interpreted in terms of a cytotoxic potentiation mechanism between ACM and DOX against K562-DOX cells. The point from Figure 4 corresponding to a simultaneous exposure with DOX (1,000 nM) and ACM (1,000 nM) ($GI_{50}$ at 1 h) is reported in Figure 5b and confirms this conclusion. In contrast the point from Figure 4 corresponding to a sequential exposure with ACM (3,000 nM) fol-

**Figure 2** Analysis of the emission spectrum from a microvolume within the nucleus of a K562 cell exposed 2 h to 1 $\mu$M DOX and 1 $\mu$M ACM. The experimental spectrum ($\Delta$) is resolved, using the minimisation of term 4 reported in the Material and methods section, into five contributions derived from these compounds: free DOX (▲), DNA-bound DOX (●), free ACM (✱), $C_l$ (■) and an untreated cell nucleus (○). The corrected fluorescence emission spectrum of DOX from a treated cell nucleus is the spectrum (□). Total intranuclear concentration of DOX, evaluated from equation (3), is 82±8 $\mu$M. Conditions of laser excitation as in Figure 1.

**Table I** Growth inhibitory concentration (GI) of DOX and ACM in K562 and K562-DOX cells

| | | *DOX* | | *ACM* | |
|---|---|---|---|---|---|
| | | *K562* | *K562-DOX* | *K562* | *K562-DOX* |
| $GI_{20}$ | (nM) | 50 | 1000 | 20 | 180 |
| $GI_{50}$ | (nM) | 80 | 2100 | 65 | 450 |
| $GI_{100}$ | (nM) | 200 | 5000 | 800 | 1400 |

Growth inhibition was determined on cells exposed for 4 h to a given drug concentration, and resuspended for 3 days in drug-free medium. Growth inhibition was calculated as per cent ratio between doubling number of treated cells and doubling number of untreated cells. Growth inhibitory concentrations were calculated from three to five independent determinations. Each standard deviation was less than 15% of the average.

**Figure 3** Growth inhibitory effect of doxorubicin on K562 and K562-DOX cells, without and with aclacinomycin A. Cells were simultaneously exposed to both drugs for 4 h at 37°C, resuspended for 3 days in drug-free medium and then counted. (□) K562 (0 nM ACM); (■) K562 (10 nM ACM); (□) K562-DOX (0 nM ACM); (■) K562-DOX (50 nM ACM). Growth inhibitory effect was calculated as in Table I. Each point represents the mean and each bar the standard deviation of three experiments. Observed differences in growth inhibitions of K562-DOX cells (■,□) are significant: $P<0.001$.

**Figure 5** Isobolograms (or isoeffect plots) of $GI_{50}$ against K562-DOX cells with ACM-DOX associations. The expected additive $GI_{50}$ are represented with calculations by mode I (■) and by mode II (□) as defined by Steel *et al.* (1979). **a**, Cells were exposed for 4 h to each drug; (●) is a projection from Figure 3 of ACM-DXR simultaneous incubation that gives $GI_{50}$. **b**, Cells were exposed for 1 h to each drug; (●) and (○) are projections from Figure 4 of simultaneous and sequential incubations respectively that give $GI_{50}$.

**Figure 4** Comparison of drug associations (ACM and DOX), on the growth inhibition of K562-DOX cells. (■) Cells were exposed simultaneously to both drugs for 1 h; (□) cells were pre-treated for 1 h with ACM, washed and resuspended for 1 h in ACM-free medium containing DOX. Cells were washed and after 3 days in drug-free medium, growth inhibitory effects were calculated. Drug concentration ratio ACM/DOX = 1. Each point represents the mean and each bar the standard deviation of three experiments.

lowed by DOX (3,000 nM) ($GI_{50}$ at 1 h) gives a point that lies within the envelope of additivity.

*Effect of ACM on the intranuclear DOX uptake*

A previous study reported that DOX cytotoxicity was dependent on the drug concentration in nuclei and that the resistant K562 subline exhibited an altered DOX uptake (Gigli *et al.*, 1989). To determine whether co-incubation with ACM produces an enhancement of DOX intranuclear concentration, we have measured intranuclear concentrations of DOX by microspectrofluorometry, as a function of extracellular doses of ACM. This relationship is shown in Figure 6. For K562-DOX cells, after 4 h of incubation with ACM and DOX (1 μM), DOX intranuclear concentrations were increased proportionally to ACM extracellular doses. In contrast, for the sensitive line, the DOX uptake into the nucleus was found to be independent of the ACM dose in the medium.

The time-course of DOX intranuclear uptake and the effect of ACM (1 μM) on this process is shown in Figure 7. The rate of DOX accumulation in the parent line is not influenced by the presence of ACM. In resistant cells, the intranuclear concentration of DOX is increased 5-fold with the presence of ACM (1 μM). During the first hour, this uptake is similar to the one of sensitive cells, but afterwards the DOX uptake reaches a maximum, contrary to the sensitive cells.

### *Effect of ACM on the intranuclear DOX retention*

It has been shown repeatedly that calcium antagonists and calmodulin inhibitors stimulate drug accumulation in resistant cells by inhibition of the drug efflux. The intranuclear retention of DOX from preloaded cells, determined by microspectrofluorometry, is shown in Figure 8. When $C_o$ is the initial intranuclear DOX concentration of preloaded cells, the DOX concentration ($C$) retained inside the nucleus in the presence of DOX-free medium is described by an exponential decrease:

$$C = C_o \,.\, 10^{-t.\log 2/T_{1/2}} \qquad (5)$$

Where $t$ is time and $T_{1/2}$ is the time corresponding to a 50% decrease of the intranuclear DOX concentration. DOX efflux from resistant cells ($T_{1/2} = 30$ min) occurs faster than that from sensitive cells ($T_{1/2} = 8$ h). The presence of ACM (1 µM) during the DOX efflux phase increased the DOX retention from K562-DOX cell nuclei ($T_{1/2} = 90$ min), but did not affect the efflux from the nuclei of sensitive cells.

A more detailed study of DOX uptake and retention will explain the weaker efficiency of sequential exposures to ACM followed by DOX, compared to simultaneous exposures to both drugs, which have been reported in the Effect of ACM on DOX-induced growth inhibition section. DOX uptakes in resistant cell nuclei are compared in Figure 9, as a function of the time, according to the type of incubation with associated DOX and ACM. DOX was permanently present in the medium, but ACM was added with various delays. The similarity of curves (◇) and (◆) shows that a pretreatment with ACM does not influence the intranuclear DOX uptake. A comparison of curves (■) and (▲) shows that a removal of ACM from the extracellular medium induces a fast DOX efflux out of the nucleus. Within 30 min the intranuclear DOX concentration has decreased by 50%, corresponding to the initial $T_{1/2}$ value (30 min) of resistant cells without ACM. However, the inhibition of DOX efflux by ACM is reversible and requires the permanent presence of ACM in the medium.

**Figure 6** DOX uptake in the nucleus of K562 (□) and K562-DOX (■) cells, as a function of extracellular concentrations of ACM. Cells were exposed simultaneously to DOX (1 µM) and ACM. After 4 h, intranuclear concentrations of DOX were determined. Vertical bars denote standard deviations on the intranuclear DOX concentration values, as determined from 30 measurements.

**Figure 7** Effect of ACM on the DOX uptake in K562 and K562-DOX cell nuclei, as a function of the time. Cells were exposed to DOX (1 µM), without and with ACM (1 µM). (□) K562 (0 µM ACM); (△) K562 (1 µM ACM); (■) K562-DOX (0 µM ACM); (▲) K562-DOX (1 µM ACM). Vertical bars denote standard deviations on the intranuclear DOX concentration values, as determined from 30 measurements.

**Figure 8** Effect of ACM on the DOX retention from nuclei of K562 and K562-DOX cells. Cells were exposed to DOX (K562, 0.5 µM; K562-DOX, 5 µM) for 1 h at 37°C. Initial intranuclear concentrations of DOX were 83 ± 13 µM and 49 ± 11 µM for K562 and K562-DOX respectively. Cells were washed with PBS and resuspended in DOX-free medium, without and with ACM (1 µM). (□) K562 (0 µM ACM); (△) K562 (1 µM ACM); (■) K562-DOX (0 µM ACM); (▲) K562-DOX (1 µM ACM). Each point represents the mean of three replicates with 30 cell nuclei. The results are given with an accuracy of 10%. Data were fitted to equation (5) with the following $T_{1/2}$: (□) 8 h; (△) 8 h; (■) 30 min; (▲) 90 min.

**Figure 9** Effects of sequential and simultaneous ACM-DOX incubations, on the intranuclear uptake of DOX in K562-DOX cells, as a function of the time. From time 0, K562-DOX cells were exposed to DOX (1 µM) at 37°C: (◇) without ACM; (◆) cells were pre-treated for 1 h with ACM (1 µM) alone, washed and resuspended at time 0 in DOX (1 µM) alone; (▲) cells were exposed for the first hour simultaneously to ACM (1 µM) with DOX, washed and resuspended in DOX alone; (■) cells were incubated for 4 h simultaneously with ACM (1 µM) and DOX. Vertical bars denote standard deviations on the intranuclear DOX concentration values, as determined from 30 measurements.

### Discussion

To avoid failure of chemotherapeutic treatment due to the emergence of drug resistance, attempts have been made to circumvent this problem either by using new drugs particularly able to overcome anthracycline resistance, or by an association of drugs where one agent increases the pharmacological effects of the other. Several classes of drugs have been shown to reverse acquired resistance to anthracycline, including calcium channel blockers (verapamil) (Tsuruo *et al.*, 1983; Friche *et al.*, 1987), calmodulin antagonists (trifluoperazine, perhexilene maleate) (Ganapathi *et al.*, 1984), triparanol analogues (tamoxifen) (Ramu *et al.*, 1984), cardiac anti-arhythmics (quinidine, amiodarone) (Chauffert *et al.*, 1986; Tsuruo *et al.*, 1984) and cyclosporins (Twentyman, 1988).

ACM, an antineoplasic agent (Umezawa *et al.*, 1987), has been shown to circumvent anthracycline resistance at the high dose of $10\,\mu g\,ml^{-1}$ (Tapiero *et al.*, 1988). Moreover, a significant synergistic effect of cytotoxicity against P388 leukaemia has been observed with the associations ACM–cyclophosphamide and ACM–vincristine (Fugimoto *et al.*, 1979). Compared with other drug combinations used to reverse the resistance, the interest in ACM is because of its own significant activity against a number of human tumours and its current use in clinical investigations (Kumai *et al.*, 1984; Majima *et al.*, 1987). Moreover, *in vitro* studies have shown a slight cross-resistance between ACM and DOX (Umezawa *et al.*, 1987; Tapiero *et al.*, 1988).

Any association of drugs is unable to restore the full sensitivity of resistant cells *in vivo*. For example, even if, *in vitro*, verapamil induces increased drug uptake in resistant cells, *in vivo* experiments indicate that verapamil, at a tolerated dose, can only partially overcome drug resistance (Friche *et al.*, 1987). This result is probably entirely due to the toxicity limitation of dosage, which allows only a insufficient plasma concentration of verapamil during the DOX uptake phase. Since our principal aim was to reverse *in vitro* drug resistance in incubation conditions close to *in vivo* conditions, we have implemented short-term incubations with ACM doses corresponding to plasma concentration from 1 μM to 200 nM during the first hour after an i.v. bolus (Egorin *et al.*, 1982). These short-term incubations probably reflect, better than continuous incubations, the *in vivo* plasma conditions following a bolus injection. In these incubation conditions, an ACM dose which alone does not produce any growth inhibition on DOX resistant cells, does induce a partial reversal of DOX resistance.

As for other agents that reverse resistance to DOX, the possible mechanism of this synergistic effect could depend on increased uptake of DOX induced by ACM in resistant cells. The mechanism of DOX action has been attributed to intercalation with DNA (Manfait *et al.*, 1982) with the result that DNA replication and RNA synthesis is inhibited (Zunino *et al.*,1980), strand-breaking of DNA by bioreductive alkylation occurs (Moore, 1977) and oxygen-free radicals are generated (Bachur *et al.*, 1979). Although the relationship between the cytotoxic effect of DOX with total cellular drug content is unclear (Lane *et al.*, 1987), we have shown previously a direct relationship between the growth inhibition effect and the DOX amount actually in the nucleus (Gigli *et al.*, 1989). Our findings suggest that there is an enhancement of DOX cytotoxicity with increased intranuclear concentration of this drug induced by ACM. The combined effect of these independent observations strongly supports the hypothesis that the nucleus is a target for DOX.

Three hypotheses could explain the increase of the amount of intranuclear DOX caused by ACM: (i) an alteration of DOX influx, (ii) an alteration of DOX efflux, or (iii) a redistribution of DOX inside the cell, allowing it to reach targets more conducive to cytotoxicity. In the light of data implicating increased activity of a membrane-associated pump called P-glycoprotein in acquired resistance, particular attention was given to drug efflux out of the nucleus in our system. With direct observation inside the nucleus of living cells, using microspectrofluorometry, we show that DOX is bound to DNA by a reversible interaction. The DOX efflux from the nucleus occurs faster in K562-DOX cells compared to sensitive cells, with the result that resistant cells are able to reduce intranuclear DOX concentrations to sublethal values by active efflux.

This study demonstrates that the presence of ACM blocks the DOX efflux in the resistant cell line, resulting in enhanced accumulation of DOX and increased cytotoxicity. Because of the rapid accumulations of ACM into sensitive and multidrug resistant lines (Seeber *et al.*, 1980; Tapiero *et al.*, 1988), the ACM intracellular deposition might in turn affect the DOX efflux. In contrast, another agent (forskolin) which reversed resistance to DOX, with increase of DOX cellular amounts, has virtually no effect on the rate of drug efflux (Wadler & Wiernik, 1988). The exact function of P-glycoprotein is not well known; however, a recent study has demonstrated drug binding to this protein (Cornwell *et al.*, 1986), suggesting its involvement in drug transport. So, the altered efflux of DOX by ACM in resistant cells could result from a molecular interaction between ACM and the P-glycoprotein or from an altered expression of this protein.

In conclusion, our results indicate that ACM partly reverses DOX-resistance at clinically achievable concentrations. We find that the reversal of resistance induced by ACM, associated with the DOX efflux inhibition, is reversible and requires the continuous presence of ACM in the medium. Thus, toxicologic, pharmacokinetic studies and clinical trials as resistance modifiers are now in progress by using the ACM-DOX association in simultaneous injections.

This work was supported in part by Association contre le cancer and by Laboratoires Roger Bellon, Paris, France. The authors are grateful to Prof. R.E. Hester for reading the manuscript, to Dr N. Austin, Dr M. Gigli and Dr P. Joly for helpful discussions and advice, to Dr J.-F. Angiboust for his help in instrument optimisation and to DILOR (Lille, France) for their contribution in instrumentation.

### References

BACHUR, N.R., GORDON, S.L., GEE, M.V. & KON, H. (1979). NADPH cytochrome P-450 reductase activation of quinone anticancer agents to free radicals. *Proc. Natl Acad. Sci. USA*, **76**, 954.

BRADLEY, G., JURANKA, P.F. & LING, V. (1988). Mechanism of multidrug resistance. *Biochim. Biophys. Acta*, **948**, 87.

CHAUFFERT, B., MARTIN, M., HAMMAN, A., MICHEL, M. & MARTIN, F. (1986). Amiodarone-induced enhancement of doxorubicin and 4'-deoxydoxorubicin cytotoxicity to rat colon cancer cells *in vitro* and *in vivo*. *Cancer Res.*, **46**, 825.

CORNWELL, M.M., GOTTESMAN, M.M. & PASTAN, I.H. (1986). Increased vinblastine binding to membrane vesicles from multidrug-resistant KB cells. *J. Biol. Chem.*, **261**, 7921.

EGORIN, M.J., VAN ECHO, D., FOX, B.M., WHITACRE, M. & BACHUR, N.R. (1982). Plasma kinetics of aclacinomycin A and its metabolites in man. *Cancer Chemother. Pharmacol.*, **8**, 41.

FRICHE, E., SKOVSGAARD, T. & NISSEN, N.I. (1987). Effect of verapamil on daunorubicin accumulation in Ehrlich ascites tumor cells. *Cancer Chemother. Pharmacol.*, **19**, 35.

FUGIMOTO, S., INAGAKI, I., HORIKOSHI, N. & OGAWA, M. (1979). Combination chemotherapy with a new anthracycline glycoside, aclacinomycin A and active drugs for malignant lymphomas in P388 mouse leukemia system. *Gann*, **70**, 411.

GANAPATHI, R., GRABOWSKI, D., ROUSE, W. & RIEGLER, F. (1984). Differential effect of the calmodulin inhibitor trifluoperazine on cellular accumulation, retention, and cytotoxicity of anthracyclines in doxorubicin (Adriamycin)-resistant P388 leukemia cells. *Cancer Res.*, **44**, 5056.

GIGLI, M., DOGLIA, S.M., MILLOT, J.-M., VALENTINI, L. & MANFAIT, M. (1988). Quantitative study of doxorubicin in living cell nuclei by microspectrofluorometry. *Biochim. Biophys. Acta*, **950**, 13.

GIGLI, M., RASOANAIVO, T.W.D., MILLOT, J.-M. & 5 others (1989). Correlation between growth inhibition and intranuclear doxorubicin and 4'-iododoxorubicin quantitated in living K562 cells by microspectrofluorometry. *Cancer Res.*, **49**, 560.

GINOT, L., JEANNESSON, P., ANGIBOUST, J.-F., JARDILLIER, J.-C. & MANFAIT, M. (1984). Interaction of adriamycin in sensitive and resistant leukemic cells, a comparative study by microspectrofluorometry. *Studia Biophys.*, **104**, 145.

KARTNER, N., RIORDAN, J.R. & LING, V. (1983). Cell surface P-glycoprotein associated with multidrug resistance in mammalian cell lines. *Science*, **221**, 1285.

KUMAI, K., KUBOTA, T., ISHIBIKI, K. & ABE, O. (1984). Experimental and clinical studies on aclarubicin in the treatment of solid tumors. *Biomed. Pharamacother.*, **38**, 332.

LANE, P., VICHI, P., BAIN, D.L. & TRITTON, T.R. (1987). Temperature dependence studies of adriamycin uptake and cytotoxicity. *Cancer Res.*, **47**, 4038.

LING, V., KARTNER, N., SUDO, T., SIMINOVITCH, L. & RIORDAN, J.R. (1983). Multidrug resistance phenotype in Chinese hamster ovary cells. *Cancer Treat. Rep.*, **67**, 869.

LOZZIO, C.B. & LOZZIO, B.B. (1975). Human chronic myelogenous leukemia cell line with positive phyladephia chromosome. *Blood*, **45**, 321.

MAJIMA, H. & OHTA, K. (1987). Clinical studies of aclacinomycin A (ACM). *Biomed. Pharmacother.*, **41**, 233.

MANFAIT, M., ALIX, A.J.P., JEANNESSON, P., JARDILLIER, J.-C. & THEOPHANIDES, T. (1982). Interaction of adriamycin with DNA as studied by resonance Raman spectroscopy. *Nucleic Acids Res.*, **10**, 3803.

MANFAIT, M., GIGLI, M., MILLOT, J.-M. & 6 others. (1987). New perspectives of microspectrofluorometry on living cells. In *Spectroscopy of Biological Molecules–New Advances*, Schmid, E.D., Schneider, F.W. & Siebert, F. (ed) p. 445. John Wiley & Sons: Chichester.

MANFAIT, M., RASOANAIVO, T., MILLOT, J.-M., ANGIBOUST, J.-F. & GIGLI, M. (1988). Pharmacokinetic and resistance to anthracyclines in living cells studied by microspectrofluorometry. In *Neoadjuvant Chemotherapy II*, Jacquillat, C., Veil, M. & Khayat, D. (ed) p. 605. John Libbey: London.

MILLOT, J.-M., RASOANAIVO, T., ANGIBOUST, J.-F., GIGLI, M. & MANFAIT, M. (1989). Microspectrofluorimétrie sur cellules vivantes: incorporation nucléaire et pharmacocinétique. *Innov. Tech. Biol. Med.*, **10**, 160.

MOORE, H.W. (1977). Bioactivation as a model for drug design bioreductive alkkylation. *Science*, **197**, 527.

OGASAWARA, T., MASUDA, Y., GOTO, S., MORI, S. & OKI, T. (1981). High performance liquid chromatographic determination of aclacinomycin A and its related compounds. II: Reverse phase HPLC determination of Aclacinomycin A and its metabolites in biological fluids using fluorescence detection. *J. Antibiot.*, **38**, 52.

RAMU, A., GLAUBIGER, D. & FUKS, Z. (1984). Reversal of acquired resistance to doxorubicin in P388 murine leukemia cells by tamoxifen and other triparanol analogues. *Cancer Res.*, **44**, 4392.

RIORDAN, J., DEUCHARS, K., KARTNER, N., ALON, N., TRENT, J. & LING. V. (1985). Amplification of P-glycoprotein gene in multidrug-resistant mammalian cell lines. *Nature*, **316**, 817.

SEEBER, S., LOTH, H. & CROOKE, S.T. (1980). Comparative nuclear and cellular incorporation of daunorubicin, doxorubicin, carminomycin, marcellomycin, aclacinomycin A and AD 32 in daunorubicin-sensitive and -resistant Ehrlich Ascites *in vitro*. *Cancer Res. Clin. Oncol.*, **98**, 109.

STEEL, G.G. & PECKHAM, M.J. (1979). Exploitable mechanisms in combined radiotherapy-chemotherapy: the concept of additivity. *Int. J. Radiat. Oncol. Biol. Phys.*, **5**, 85.

SUGIMOTO, Y. & TSURUO, T. (1987). DNA-mediated transfer and cloning of a human multidrug-resistant myelogeneous leukemia K562. *Cancer Res.*, **47**, 2620.

TAPIERO, H., BOULE, D., TRINCAL, G., FOURCADE, A. & LAMPIDIS, T.J. (1988). Potentiation of ADM accumulation and effectiness in ADM-resistant cell by aclacinomycin A. *Leukemia Res.*, **12**, 411.

TSURUO, T., IIDA, H., NOJIR, M., TSUKAGOSHI, S. & SAKURAI, Y. (1983). Circumvention of vincristine and adriamycin resistance *in vitro* and *in vivo* by calcium influx blockers. *Cancer Res.*, **43**, 2905.

TSURUO, T., IIDA, H., KATANI, Y., YOKOTA, K., TSUKAGOSHI, S. & SAKURAI, Y. (1984). Effects of quinidine and related compounds on cytotoxicity and cellular accumulation of vincristine and adriamycin in drug-resistant tumour cells. *Cancer Res.*, **44**, 4303.

TSURUO, T., IIDA, H., KAWATABA, H., OH-HARA, T., HAMADA, H. & UTAKOJI, T. (1986). Characteristics of resistance to adriamycin in human myelogenous leukemia K562 resistant to adriamycin and in isolated clones. *Jpn. J. Cancer Res.*, **77**, 682.

TWENTYMAN, P.R. (1988). Modification of cytotoxic drug resistance by non-immuno-suppressive cyclosporins. *Br. J. Cancer*, **57**, 254.

UMEZAWA, K., KUNIMOTO, S. & TAKEUCHI, T. (1987). Experimental studies of new anthracyclines: aclacinomycin, THP adriamycin and ditrisarubicins. *Biomed. Pharmacother.*, **41**, 206.

WADLER, S. & WIERNIK, P.H. (1988). Partial reversal of doxorubicin resistance by forskolin and 1,9-dideoxyforskolin in murine sarcoma S180 variants. *Cancer Res*, **48**, 539.

ZUNINO, F., DIMARCO, A., ZACCARA, A. & GAMBETTA, R.A. (1980). The interaction of daunorubicin and doxorubicin with DNA and chromatin. *Biochim. Biophys. Acta*, **607**, 206.